# Time trends in primary therapy and relative survival of diffuse large B-cell lymphoma by stage: a nationwide, population-based study in the Netherlands, 1989–2018

**DOI:** 10.1038/s41408-022-00637-1

**Published:** 2022-03-09

**Authors:** Müjde Durmaz, Otto Visser, Eduardus F. M. Posthuma, Rolf E. Brouwer, Djamila E. Issa, Daphne de Jong, King H. Lam, Nicole M. A. Blijlevens, Josée M. Zijlstra, Martine E. D. Chamuleau, Pieternella J. Lugtenburg, Marie José Kersten, Avinash G. Dinmohamed

**Affiliations:** 1 Department of Research and Development, Netherlands Comprehensive Cancer Organisation (IKNL), Utrecht, The Netherlands; 2 Department of Registration, Netherlands Comprehensive Cancer Organisation (IKNL), Utrecht, The Netherlands; 3 Department of Internal Medicine, Reinier de Graaf Gasthuis, Delft, The Netherlands; 4 Department of Hematology, Leiden University Medical Center, Leiden, The Netherlands; 5 Department of Internal Medicine, Jeroen Bosch Hospital, Den Bosch, The Netherlands; 6 Amsterdam UMC, Department of Pathology, Vrije Universiteit Amsterdam, Cancer Center Amsterdam, Amsterdam, The Netherlands; 7 Department of Pathology, Erasmus MC Cancer Institute, Rotterdam, The Netherlands; 8 Department of Hematology, Radboud University Medical Center, Nijmegen, The Netherlands; 9 Amsterdam UMC, Vrije Universiteit Amsterdam, Cancer Center Amsterdam, Department of Hematology, Amsterdam, The Netherlands; 10 Department of Hematology, Erasmus MC, Cancer Institute, University Medical Center Rotterdam, Rotterdam, The Netherlands; 11 Amsterdam UMC, University of Amsterdam, Department of Hematology, Amsterdam, The Netherlands; 12 Department of Public Health, Erasmus MC, University Medical Center Rotterdam, Rotterdam, The Netherlands

**Keywords:** Cancer epidemiology, B-cell lymphoma

## Abstract

It is unclear whether survival in diffuse large B-cell lymphoma (DLBCL) continues to increase in an era where rituximab-containing chemotherapy reigns for almost two decades. Therefore, we evaluated trends in primary therapy and relative survival (RS) among Dutch DLBCL patients diagnosed between 1989 and 2018. Analyses were performed separately according to the stage I (*N* = 6952) and stage II–IV disease (*N* = 20,676), stratified by calendar period and age (18–64, 65–74, and ≥75 years). The use of chemotherapy ± radiotherapy increased over time across all age and stage groups. As of the mid-2000s, >95% of chemotherapy-treated patients received chemoimmunotherapy, irrespective of age and stage. Overall, RS increased significantly over time across all age groups, especially after 2003 when rituximab-containing chemotherapy had become the standard of care. However, RS increased less pronounced between 2003–2010 and 2011–2018 than between 1989–2002 and 2003–2010. These findings were congruent across all studied stage groups. Five-year RS across the three age groups during 2011–2018 was 96%, 84%, and 67% for stage I DLBCL and 75%, 60%, and 46% for stage II–IV DLBCL. Collectively, survival in DLBCL increased modestly beyond the initial introduction of rituximab, with apparent survival differences across age and stage that warrant novel treatment approaches.

## Introduction

Diffuse large B-cell lymphoma (DLBCL) is the most common type of non-Hodgkin lymphoma (NHL), accounting for approximately one-third of all newly diagnosed NHL cases in Western countries [1, 2]. DLBCL is a heterogeneous lymphoma with significant variation in clinical and biologic characteristics, response to therapy, and prognosis [3–5]. The age-standardized incidence rate (ASR) of DLBCL in Western countries approximates 6–7 per 100,000 person-years, with higher rates in males and individuals over 65 years of age [1, 6, 7].

The addition of rituximab to the chemotherapy regimen consisting of cyclophosphamide, doxorubicin, vincristine, and prednisone (R-CHOP) dramatically changed the treatment paradigm of DLBCL in the early 2000s [8–10]. Depending on patients’ age, the disease stage and biology, around 40–70% of patients can be cured with first-line R-CHOP [8–11].

At present, the almost 2-decades-old R-CHOP regimen is still the standard of care for patients with DLBCL in the first line. Novel combinations (e.g., adding lenalidomide, bortezomib, or ibrutinib) to this regimen have not been shown to dramatically outperform R-CHOP [12–15]. Notwithstanding, progress has been made by augmenting supportive care measures, improving risk-adapted therapy (e.g., through refining the classification system of lymphomas), and optimizing the interval and number of cycles of R-CHOP [16–22].

Population-based studies demonstrated that the implementation of R-CHOP into routine clinical practice had significantly improved the population-level survival of patients with DLBCL. However, these studies primarily covered the first decade of 2000 [7, 23–26], with only one study reporting overall stage-specific survival patterns of patients with DLBCL diagnosed between 2002 and 2013 [27]. Therefore, this nationwide, population-based study addressed the current knowledge gap by focussing on stage-specific patterns in primary therapy and survival across different age groups and calendar periods in the Netherlands. Besides, we were interested in ascertaining whether the survival of adult patients with DLBCL continued to increase in contemporary clinical practice.

## Patients and methods

### The Netherlands Cancer Registry

Nationwide since 1989, the Netherlands Cancer Registry (NCR) covers more than 95% of all newly diagnosed malignancies in the Netherlands [28]. The NCR builds on comprehensive case notification by all Dutch pathology laboratories through the Nationwide Network and Registry of Histopathology and Cytopathology and the National Registry of Hospital Discharges (i.e., inpatient and outpatient discharges). After case notification, trained NCR registrars collect basic details through retrospective medical records review on patient- (e.g., sex and dates of birth and diagnosis) and tumor characteristics (e.g., disease stage and topography and morphology codes according to classification system valid at the time of DLBCL diagnosis) and primary therapy. Therapy after disease progression was not registered. Tumor topography and morphology are coded according to the International Classification of Diseases for Oncology (ICD-O). Information on patients’ vital status (i.e., alive, dead, or emigration) was obtained via annual linkage with the Nationwide Population Registries Network that holds this information of all residents in the Netherlands.

### Study population

We identified all patients diagnosed with primary DLBCL between January 1, 1989, and December 31, 2018—with survival follow-up through January 1, 2021—from the NCR using ICD-O morphology codes; 9593, 9675, 9680, 9681, 9682, and 9684. We excluded patients with primary central nervous system lymphoma and primary mediastinal B-cell lymphoma. Further, patients <18 years at diagnosis (*n* = 193) and those diagnosed at autopsy (*n* = 153) were excluded. However, these two groups were not excluded from the analysis to calculate the overall incidence rate. This approach is congruent with international standards for calculating overall incidence rates. All patients were followed for survival from the date of diagnosis to death, emigration, or end of follow-up (i.e., January 1, 2021), whichever occurred first.

According to the Central Committee on Research involving Human Subjects (CCMO), this type of observational, non-interventional study does not require approval from an ethics committee in the Netherlands. The Privacy Review Board of the NCR approved the use of anonymous data for this study.

### Primary therapy

The categories for primary therapy were defined as follows: (i) no anti-neoplastic therapy, (ii) radiotherapy alone, (iii) chemotherapy without radiotherapy, (iv) chemotherapy with radiotherapy (i.e., combined modality treatment; CMT), and (v) other/unknown therapy. Primary therapy was presented for three calendar periods (1989–2002, 2003–2010, and 2011–2018) according to three age groups at diagnosis (18–64, 65–74, and >75 years), stratified by disease stage as per the Ann Arbor classification (stage I and II–IV). Of note, information on bulky disease was not available in the NCR. The first epoch represents the pre-rituximab era. The second and third epochs represent the era in which rituximab-containing chemotherapy was gradually implemented into daily practice and in which rituximab-containing chemotherapy was considered the standard first-line therapy, respectively.

Information on rituximab use was recorded in the NCR for patients diagnosed as from January 1, 2007. The results on chemoimmunotherapy with rituximab were presented as the proportion of rituximab within the group of chemotherapy-treated patients. These results were presented according to age and stage categories as described previously.

The NCR ascertains more detailed information on the exact first-line treatment regimens for patients diagnosed as of January 1, 2014. The type of regimens for patients diagnosed during 2014–2018 was categorized as R-CHOP every 21 (R-CHOP21) or 14 days (R-CHOP14), rituximab ± other, less commonly applied agents, radiotherapy alone, other/unknown therapy, and no anti-neoplastic therapy. Furthermore, treatment with R-CHOP was subdivided according to the number of treatment cycles for the most commonly applied schedules, namely eight cycles of R-CHOP21 (8× R-CHOP21), six cycles of R-CHOP21 (6× R-CHOP21), 6× R-CHOP21 with two additional rituximab cycles (6× R-CHOP21 + 2 R), six cycles of R-CHOP14 (6× R-CHOP14) and three cycles of R-CHOP21 plus radiotherapy (3× R-CHOP21 + RT). Treatment schemes with different R-CHOP intervals and cycles were grouped into a separate categories. The exact therapeutic regimens are presented for the three earlier described age groups according to disease stage, stratified for each calendar year of diagnosis (i.e., from 2014 to 2018), unless otherwise stated. Lastly, information was available on the reasons why anti-neoplastic therapy was not started.

### Statistical analyses

Descriptive statistics were used to delineate patient and treatment features. We used the Pearson chi-square test to compare categorical variables and the Kruskal–Wallis test to compare continuous variables.

Incidence rates of DLBCL were computed per 100,000 person-years using the annual mid-year population size obtained from Statistics Netherlands. The overall and sex-specific incidence rates were age-standardized as per the European standard population to account for the varying age structures and compare these rates across different populations or over time. Incidence rates were calculated overall and stratified by sex, age (20–64, 65–74, and ≥75 years), and calendar period. These rates were also presented separately according to disease stage. Besides, age-specific incidence rates were calculated for quinquennial years of age (i.e., from 0–5 years to ≥90 years) overall and according to sex and disease stage. The classification of the age-specific incidence rates slightly differs from the age categories defined previously because this quinquennial classification is frequently applied in the literature to compare incident rates internationally.

Linear trends in the application of primary therapy with each successive calendar period were assessed using a nonparametric test of the trend for ranks across ordered groups, which is an extension of the Wilcoxon rank-sum test.

We computed relative survival to estimate the disease-specific survival because information on the cause of death was not available in the NCR [28]. Relative survival is calculated as the ratio of patients’ overall survival and expected survival of an equivalent sex-, age-, and calendar period-matched group from the general population [29]. Thus, relative survival portrays the excess mortality—relative to the general population’s mortality—related to a cancer diagnosis, regardless of whether the excess mortality was directly or indirectly ascribed to the cancer diagnosis [30]. The Ederer II methodology was used to estimate the general population’s expected survival using Dutch population life tables, stratified by age, sex, and calendar year [31]. Relative survival rates (RSRs)—with associated 95% confidence intervals (CIs) for the projected 5- and 10-year RSRs—were calculated up to ten years post-diagnosis for three calendar periods according to three age categories (18–64, 65–74, and ≥75 years), stratified by disease stage.

We assessed linear trends in RSRs over the calendar periods studied according to three age groups using Poisson regression. Also, we used Poisson regression to model excess mortality over the calendar periods studied during the first five years after DLBCL diagnosis stratified by age, with concurrent adjustment for sex, disease stage, and years of follow-up (split into 1-year time bands) [30]. The model produces excess mortality rate ratios (EMRRs), with associated 95% CIs, and was separately built according to disease stage. The calendar period 2003–2010 was selected as the reference since we aimed to assess whether excess mortality decreased in the most recent calendar period (2011–2018).

A *P* value of less than 0.05 was considered statistically significant. All statistical analyses were executed using STATA/SE 16.1 (StataCorp, TX, USA).

## Results

### Patient characteristics

Our analytic cohort included 29,067 adult (≥18 years) patients diagnosed with DLBCL (54% males; median age, 69 years) in the Netherlands between 1989 and 2018. Patient characteristics are presented in Table 1 according to the calendar period of diagnosis, stratified by disease stage. Overall, females were younger than males at diagnosis (median age, 67 versus 71 years; *P* < 0.001). Most patients were diagnosed with stage II–IV disease (71%), followed by stage I disease (24%) and unknown disease stage (5%). The proportion of patients with an unknown disease stage decreased from 8 to 3% between 1989–2002 and 2011–2018 (*P* < 0.001). Furthermore, the proportion of stage I decreased from 28 to 19%. Consequently, the proportion of stage II–IV increased from 63 to 79%. The median age at diagnosis among patients with stage I and stage II–IV disease was similar (68 versus 68 years; *P* = 0.669). Lastly, most patients with an advanced disease stage (i.e., stage II–IV) had stage IV disease (46%).

**Table 1:** Patient characteristics.

Stage	Characteristics	Calendar period						Total	
		1989–2002		2003–2010		2011–2018			
		*N*	(%)	*N*	(%)	*N*	(%)	*N*	(%)
Total	Total no. of patients	10,911		8350		9806		29,067	
	Sex								
	Male	5768	(53)	4509	(54)	5551	(57)	15,828	(54)
	Female	5143	(47)	3841	(46)	4255	(43)	13,239	(46)
	Age, years								
	Median (IQR)	68 (54–76)		69 (58–78)		69 (59–77)		69 (57–77)	
	18–64	4679	(43)	3300	(40)	3526	(36)	11,505	(40)
	65–74	2888	(26)	2128	(25)	2946	(30)	7962	(27)
	≥75	3344	(31)	2922	(35)	3334	(34)	9600	(33)
	Disease stage								
	I	3093	(28)	2027	(24)	1832	(19)	6952	(24)
	II–IV	6922	(63)	6039	(72)	7715	(79)	20,676	(71)
	Unknown	896	(8)	284	(3)	259	(3)	1439	(5)
I	Total no. of patients	3093		2027		1832		6952	
	Sex								
	Male	1637	(53)	1113	(55)	1055	(58)	3805	(55)
	Female	1456	(47)	914	(45)	777	(42)	3147	(45)
	Age, years								
	Median (IQR)	67 (53–77)		69 (58–78)		69 (59–78)		68 (56–78)	
	18–64	1376	(44)	802	(40)	669	(37)	2847	(41)
	65–74	771	(25)	491	(24)	511	(28)	1773	(26)
	≥75	946	(31)	734	(36)	652	(36)	2332	(34)
II–IV	Total no. of patients	6922		6039		7715		20,676	
	Sex								
	Male	3688	(53)	3258	(54)	4370	(57)	11,316	(55)
	Female	3234	(47)	2781	(46)	3345	(43)	9360	(45)
	Age, years								
	Median (IQR)	67 (54–76)		68 (57–77)		69 (59–77)		68 (57–77)	
	18–64	3072	(44)	2451	(41)	2825	(37)	8348	(40)
	65–74	1862	(27)	1586	(26)	2392	(31)	5840	(28)
	≥75	1988	(29)	2002	(33)	2498	(32)	6488	(31)
	Disease stage								
	II	2397	(35)	1844	(31)	1879	(24)	6120	(30)
	III	1609	(23)	1678	(28)	1828	(24)	5115	(25)
	IV	2916	(42)	2517	(42)	4008	(52)	9441	(46)

*IQR* interquartile range.

### Incidence

The overall ASR increased from 4.73 to 5.30 per 100,000 person-years between 1989–2002 and 2003–2010 (Supplemental Table 1). Thereafter, it remained comparatively stable (i.e., 5.36 per 100,000 person-years during 2011–2018). The initial increase was primarily objectified in patients with stage II–IV disease, irrespective of sex and age (Supplemental Table 1). Nonetheless, the increase was most pronounced among patients aged ≥65 years. The incidence of stage I disease decreased to some extent between 2003–2010 and 2011–2018, particularly among patients aged ≥75 years (Supplemental Table 1). The male predominance in incidence prevailed over time across all age groups and disease stages (Supplemental Fig. 1). In the most recent calendar period (i.e., 2011–2018), the incidence increased markedly after the age of 50 and peaked in the eighth decade of life. This phenomenon was independent of sex and disease stage (Supplemental Fig. 2).

### Primary treatment of stage I disease

**Fig. 1:**
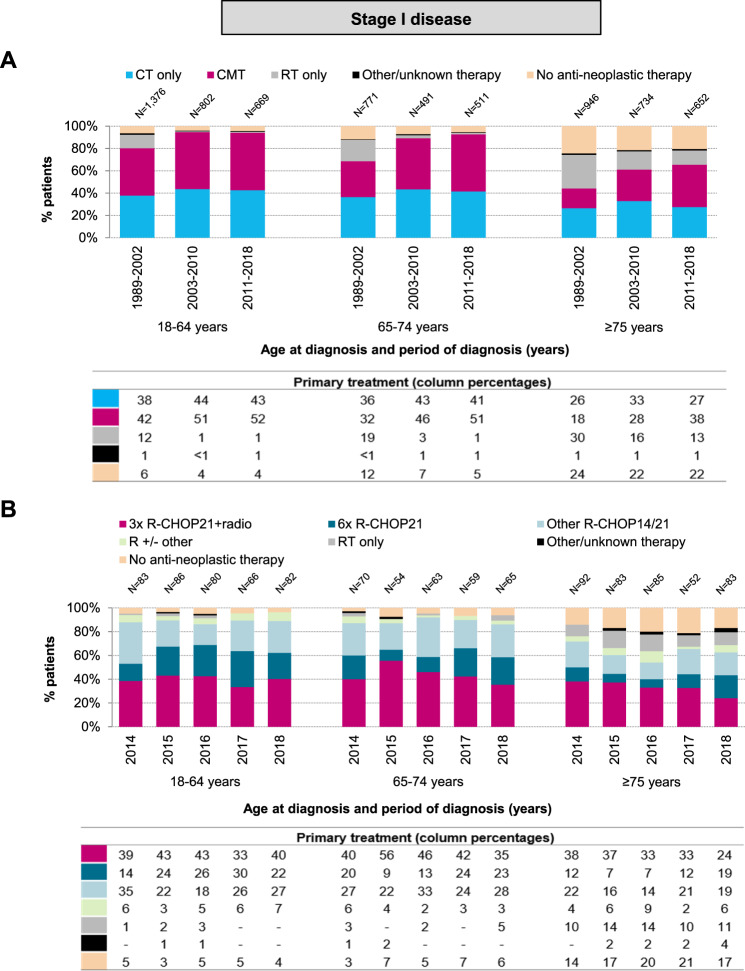
Primary treatment of adult patients diagnosed with stage I DLBCL in the Netherlands. **A** shows the results of primary therapy in broad categories according to age at diagnosis and calendar period of diagnosis for patients diagnosed during the calendar period 1989–2018. **B** shows the specific type of primary therapy according to age at diagnosis and calendar year of diagnosis for patients diagnosed between 2014 and 2018. The proportion of patients receiving a particular treatment within a specific calendar period or year and age group are presented in the column below. CT chemotherapy, CMT combined modality treatment, RT radiotherapy, R rituximab, CHOP cyclophosphamide, doxorubicin, vincristine, and prednisone.

The distribution of primary therapy among patients with stage I DLBCL according to age at diagnosis and calendar period of diagnosis is presented in Fig. 1A. Overall, CMT was the most frequently applied therapy among patients aged 18–64 and 65–74 years. Its application increased over time across all age groups (*P*_trend_ < 0.001 for all comparisons), especially between 1989–2002 and 2003–2010. Thereafter, the increase was restricted to patients aged ≥75 years (*P*_trend_ < 0.001). The proportions of CMT across the three age groups were 51%, 46%, and 28% in 2003–2010 compared to 52%, 51%, and 38% in 2011–2018. As for the application of chemotherapy without radiotherapy, there were no noteworthy increases in its application between 2003–2010 and 2011–2018 across the three age groups (43%, 41%, and 27% during 2011–2018, respectively; *P*_trend_ > 0.05 for all comparisons). Furthermore, the proportion of patients not receiving therapy was consistently higher throughout the study period in patients aged ≥75 years. Radiotherapy alone was rarely applied in the rituximab era (2003–2018) compared to the pre-rituximab era (1989–2002), especially among patients aged 18–64 and 65–74 years.

Approximately 96% of the chemotherapy-treated patients received chemoimmunotherapy during 2007–2018, irrespective of age. Furthermore, a detailed analysis of 1103 patients diagnosed during 2014–2018 (patient characteristics presented in Supplemental Table 2) showed that most chemotherapy-treated patients across the three age groups received R-CHOP21 (Supplemental Fig. 3). Conversely, R-CHOP14 was rarely applied (Supplemental Fig. 3). Regarding the number of R-CHOP cycles, 3× R-CHOP21 + RT was the most frequently applied regimen across the three age groups (40%, 43%, and 33% during 2014–2018; Fig. 1B), followed by 6× R-CHOP21 (23%, 18%, and 11% during 2014–2018; Fig. 1B).

Of the 103 patients (median age 79 years; interquartile range, 71–86 years) who received no anti-neoplastic therapy, the following reasons to refrain from it were retrievable in the medical records: patient or family member refusal (*n* = 29), patient frailty (*n* = 16), initial watch-and-wait approach (*n* = 14), comorbidities (*n* = 14), lymph node extirpation only (*n* = 9), short anticipated life expectancy (*n* = 3), rapidly progressive disease (*n* = 2), and some combination of the reasons as mentioned above (*n* = 4). In the remaining ten patients, the reasons were unknown.

### Primary treatment of stage II–IV disease

**Fig. 2:**
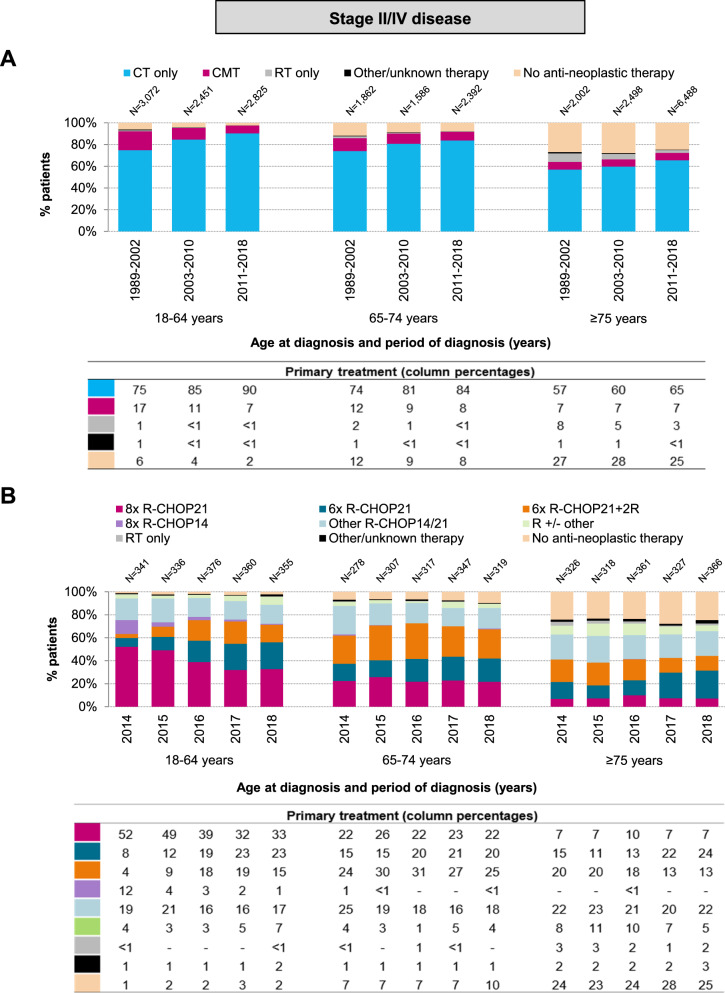
Primary treatment of adult patients diagnosed with stage II–IV DLBCL in the Netherlands. **A** shows the results of primary therapy in broad categories according to age at diagnosis and calendar period of diagnosis for patients diagnosed during the calendar period 1989–2018. **B** shows the specific type of primary therapy according to age at diagnosis and calendar year of diagnosis for patients diagnosed between 2014 and 2018. The proportion of patients receiving a particular treatment within a specific calendar period or year and age group are presented in the column below. CT chemotherapy, CMT combined modality treatment, RT radiotherapy, R rituximab, CHOP cyclophosphamide, doxorubicin, vincristine, and prednisone.

The distribution of primary therapy among patients with stage II–IV DLBCL according to age at diagnosis and calendar period is presented in Fig. 2A. Compared to patients with stage I DLBCL, chemotherapy without radiotherapy was the most frequently applied therapy among patients with stage II–IV DLBCL across all age groups. The use of chemotherapy without radiotherapy increased over time across all age groups (*P*_trend_ < 0.001 for all comparisons). Furthermore, similar to patients with stage I DLBCL, the overall use of chemotherapy was lower among patients aged ≥75 years than their younger counterparts.

During 2007–2018, more than 95% of the chemotherapy-treated patients across the three age groups received chemoimmunotherapy. Detailed information of patients diagnosed during 2014–2018 (patient characteristics presented in Supplemental Table 2) showed that most patients across the three age groups received R-CHOP21 (Supplemental Fig. 4). The application of R-CHOP14 was comparatively low and primarily restricted to patients aged 18–64 and 65–74 years, of which its use gradually decreased over time (Supplemental Fig. 4). Regarding the number of treatment cycles, patients aged 18–64 years were most frequently managed with 8× R-CHOP21 (41%) compared to patients aged 65–74 (23%) and ≥75 years (8%; Fig. 2B). The latter two age groups were primarily managed with 6× R-CHOP ± two additional rituximab cycles (Fig. 2B). Interestingly, the use of 8× R-CHOP21 in patients aged 18–64 years decreased over time (from 52 to 33% between 2014 and 2018), following a broader use of 6× R-CHOP21 ± two additional rituximab cycles (from 12 to 38% between 2014 and 2018; Fig. 2B).

Overall, 573 patients (median age 80 years; interquartile range, 74–85 years) did not receive anti-neoplastic therapy. The following reasons were reported to refrain from it: patient frailty (*n* = 226), patient or family member refusal (*n* = 138), short anticipated life expectancy (*n* = 62), comorbidities (*n* = 44), rapidly progressive disease (*n* = 36), watch-and-wait approach (*n* = 9), lymph node extirpation only (*n* = 1), other, unspecified reasons (*n* = 1), and some combination of the reasons as mentioned above (*n* = 16). In 40 patients, the reasons were unknown”.

### Relative survival of stage I disease

**Fig. 3:**
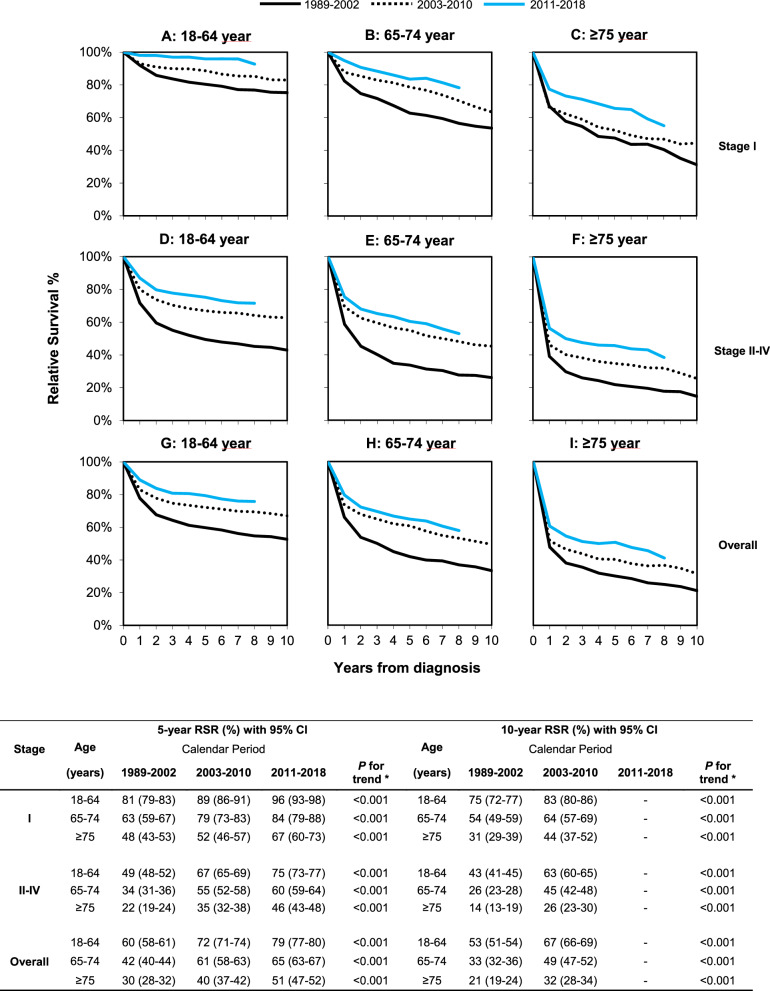
Relative survival of adult patients diagnosed with DLBCL in the Netherlands according to age at diagnosis and calendar period of diagnosis. Relative survival of patients with stage I disease is presented according to the following age categories: **A** 18–64, **B** 65–74, and **C** ≥ 75 years. The corresponding relative survival of patients with stage II–IV disease is shown in panels **D** to **F**. Lastly, relative survival for all stages combined is displayed in panels **G** to **I**. The table presents the projected 5- and 10-year relative survival rates with 95% confidence intervals according to age at diagnosis and calendar period of diagnosis. The asterisk indicates the *P* value for the likelihood ratio test assessing linear trends in relative survival over the calendar periods studied.

Relative survival of patients with stage I DLBCL according to age at diagnosis and calendar period of diagnosis is shown in Fig. 3A–C. Overall, 5- and 10-year relative survival improved over time across all age groups. This improvement was most pronounced among patients aged 65–74 years between 1989–2002 and 2003–2010. Thereafter, the increase was more gradual. The improvement was most pronounced for patients aged ≥75 years between 2003–2010 and 2011–2018.

The age-stratified multivariable model for relative survival corroborates an improvement of relative survival during 2011–2018 compared with 2003–2010 across all groups (Table 2). This model also shows an adverse prognostic effect of older age, but not sex.

**Table 2:** Excess mortality rate ratio (EMRR) during the first 5 years after diagnosis of stage I and stage II–IV diffuse large B-cell lymphoma in the Netherlands, 1989–2018.

Age (years)	Covariate	Stage I			Stage II–IV			Overall		
		EMRR^a^	95% CI	*P* value^b^	EMRR^a^	95% CI	*P* value^b^	EMRR^a^	95% CI	*P* value^b^
18–64	Period of diagnosis									
	1989–2002	1.66	1.29–2.14	<0.001	1.75	1.61–1.91	<0.001	1.75	1.61–1.90	<0.001
	2003–2010	1	Reference		1	Reference		1	Reference	
	2011–2018	0.29	0.16–0.51	<0.001	0.66	0.59–0.74	<0.001	0.64	0.57–0.71	<0.001
	Sex									
	Male	1	Reference		1	Reference		1	Reference	
	Female	1.18	0.95–1.47	0.129	0.89	0.82–0.96	0.002	0.91	0.85–0.98	0.013
	Stage									
	I	–	–	–	–	–	–	1	Reference	
	II	–	–	–	1	Reference		2.04	1.78–2.34	<0.001
	III	–	–	–	1.72	1.54–1.93	<0.001	3.52	3.08–4.03	<0.001
	IV	–	–	–	2.54	2.30–2.79	<0.001	5.20	4.62–5.87	<0.001
65–74	Period of diagnosis									
	1989–2002	1.78	1.38–2.31	<0.001	1.73	1.57–1.91	<0.001	1.75	1.59–1.91	<0.001
	2003–2010	1	Reference		1	Reference		1	Reference	
	2011–2018	0.61	0.42–0.89	0.010	0.75	0.68–0.84	<0.001	0.74	0.67–0.82	<0.001
	Sex									
	Male	1	Reference		1	Reference		1	Reference	
	Female	0.84	0.68–1.04	0.117	0.86	0.79–0.93	<0.001	0.86	0.79–0.92	<0.001
	Stage									
	I	–	–	–	–	–	–	1	Reference	
	II	–	–	–	1	Reference		1.76	1.52–2.03	<0.001
	III	–	–	–	1.47	1.30–1.67	<0.001	2.59	2.26–2.98	<0.001
	IV	–	–	–	2.16	1.94–2.40	<0.001	3.81	3.37–4.31	<0.001
≥75	Period of diagnosis									
	1989–2002	1.03	0.87–1.22	0.718	1.32	1.21–1.43	<0.001	1.26	1.18–1.36	<0.001
	2003–2010	1	Reference		1	Reference		1	Reference	
	2011–2018	0.63	0.51–0.77	<0.001	0.70	0.65–0.76	<0.001	0.69	0.64–0.75	<0.001
	Sex									
	Male	1	Reference		1	Reference		1	Reference	
	Female	1.19	1.03–1.39	0.022	1.01	0.94–1.08	0.868	1.03	0.97–1.10	0.316
	Stage									
	I	–	–	–	–	–	–	1	Reference	
	II	–	–	–	1	Reference		1.57	1.43–1.74	<0.001
	III	–	–	–	1.42	1.29–1.56	<0.001	2.23	2.02–2.47	<0.001
	IV	–	–	–	1.80	1.65–1.95	<0.001	2.83	2.59–3.09	<0.001

*EMR* excess mortality ratio, *DLBCL* diffuse large B-cell lymphoma, *CI* confidence interval.

^a^Each covariate is simultaneously adjusted for all other covariates in the table, along with five years of follow-up.

^b^*P* values are compared with the reference category.

### Relative survival of stage II–IV disease

Relative survival of patients with stage II–IV DLBCL according to age at diagnosis and calendar period of diagnosis is shown in Fig. 3D–F. The improvement in relative survival was generally more conspicuous over time for patients with stage II–IV DLBCL than for patients with stage I DLBCL, especially between 1989–2002 and 2003–2010. Thereafter, similar to the observations in stage I DLBCL, the improvement was more gradual.

The age-stratified multivariable model for relative survival substantiates that the relative survival improved during 2011–2018 compared with 2003–2010 (Table 2). This model also shows that sex, older age, and higher stage were poor prognostic factors.

### Additional survival analyses for all disease stages combined

Lastly, we assessed relative survival according to age at diagnosis and calendar period of diagnosis for all disease stages combined (Fig. 3H–I). This endeavor was undertaken to disaffirm that the survival improvement according to disease stage is induced by stage migration. This analysis shows that relative survival increased over time across all age groups, suggesting that stage migration only marginally affected the survival estimates. The age-stratified multivariable model of relative survival attests to these findings, as well as an adverse prognostic effect of the male sex, older age, and advanced stage (Table 2).

## Discussion

This nationwide, population-based study is the first that comprehensively assessed time trends in incidence, primary therapy, and relative survival among adult DLBCL patients according to various age groups and disease stages from a historical and contemporary perspective. This study shows differences in the application of primary treatment and improvements in relative survival in an era where first-line treatment with rituximab-based regimens reigns for almost two decades.

### Incidence

The incidence of stage II–IV DLBCL gradually increased over time, following a slight decrease of stage I disease as of the mid-2000. This finding is congruent with recent epidemiological studies in other lymphomas [32, 33]. The gradual implementation of PET-CT in the early 2000s likely explains this phenomenon, resulting in stage migration and a decrease of unstaged patients because PET-CT more accurately detects nodal and extranodal lesions than CT alone [34, 35].

The modern-day ASR in our study was somewhat lower than the ASR reported in other countries [1, 6, 27, 36], particularly when compared to Central European countries [37]. This variation might be attributed to geographical differences in lymphoma etiology (e.g., higher incidence of HIV and different genetic and environmental backgrounds) [37, 38]. Moreover, different registration practices and the type of age-standardization used to account for the differences in the age structure of the populations being compared might also explain the variation in incidence rates across countries [1, 6, 27, 36].

### Primary therapy

Randomized trials in the pre-rituximab era showed that abbreviated CMT—i.e., 3× CHOP plus involved-field radiotherapy (IFRT)—is more effective than extended chemotherapy to manage limited-stage DLBCL [36, 39–41]. The addition of rituximab to abbreviated CMT further improved disease control [42]. CMT was generally the preferred choice in the Netherlands to manage patients with stage I DLBCL, of which its application gradually increased over time. Our study’s detailed treatment data showed that 3× R-CHOP21 + RT was favored over 6× R-CHOP21. The choice for abbreviated chemoimmunotherapy with radiotherapy or extended chemoimmunotherapy without radiotherapy in limited-stage DLBCL depends on several factors, including the patient (e.g., age and comorbidity) and lymphoma characteristics (e.g., disease extension and localization), patient preference (e.g., a short or long course of treatment), and expected short- and long-term toxicities (e.g., cardiac disease and second primary malignancies) [41, 43–45]. Modern innovations with (i) reducing the radiation dose [46], (ii) replacing IFRT with involved-node radiotherapy [47, 48], (iii) improvement of radiation techniques [35], (iv) PET-guided omission of radiotherapy [49, 50], (v) reducing the chemotherapy cycles of extended chemoimmunotherapy [51], and (vi) omitting the two additional cycles of rituximab after 6× R-CHOP [52] are associated with a more favorable toxicity profile, without seemingly compromising the outcome. Moreover, the current population-based study serves as a benchmark to assess how 4× R-CHOP21 (±2R) without RT will be adopted in Dutch clinical practice to manage contemporary diagnosed patients with limited-stage DLBCL, following the results of the randomized, phase 3, non-inferiority FLYER trial and the broader utilization of PET-guided treatment [51].

Patients with stage II–IV DLBCL were primarily managed with chemotherapy without radiotherapy, aligned with clinical practice guidelines for patients with advanced-stage DLBCL [53, 54].

Our study’s detailed treatment data demonstrated a decline in the use of R-CHOP14 and increased use of R-CHOP21. This turning point likely results from several studies showing that both treatment modalities are equally effective regarding survival outcomes, with R-CHOP21 being associated with less toxicity and better health-related quality of life aspects compared to R-CHOP14 [17, 18, 55, 56]. Similarly, several studies demonstrated that 6× and 8× R-CHOP are equally effective to manage patients with advanced-stage DLBCL, with the former resulting in less toxicity [12, 15, 18, 19, 52, 57]. Following these observations, 8× R-CHOP21 was less often applied over time in the Netherlands, following a broader application of 6× R-CHOP21—particularly among patients aged 18–64 years. Furthermore, the PETAL study demonstrated that the two additional administrations of rituximab after 6× R-CHOP could be omitted in interim-PET negative patients with advanced-stage DLBCL [58]. Forthcoming population-based research is warranted to assess how this novel treatment practice will be adopted in Dutch clinical practice.

### Relative survival

The addition of rituximab to CHOP dramatically improved survival outcomes, as shown in clinical trials [8–11, 52, 59, 60] and population-based studies [23–26, 42, 55]. However, current population-based studies primarily spanned a period of 5–10 years following the introduction of rituximab in clinical practice. Therefore, it was unclear whether survival in DLBCL continues to increase in an era where rituximab-containing chemotherapy reigns for almost two decades. We addressed this knowledge gap by focussing on DLBCL patients diagnosed in the Netherlands between 1989 and 2018 with survival follow-up until January 1, 2021.

We observed that relative survival of adult DLBCL patients across all age and stage groups continue to improve significantly, albeit modestly, in a realm where novel therapeutic approaches did not substantially outperform first-line treatment with R-CHOP. This finding was congruent with a recent population-based study from the US among DLBCL patients diagnosed between 2000 and 2013 with survival follow-up through 2016 [27]. However, that study lacked data on treatment, and outcomes according to disease stage were not stratified by age.

The information gleaned from our study and the prior referenced study from the US confirms that outcomes can still improve, albeit modestly, beyond the introduction of rituximab [27]. This proposition warrants brief consideration through various prisms. First, progress in reducing the radiation field size [46], reducing dose intensity and dose interval of R-CHOP [51, 52], omitting radiotherapy [49], and augmenting supportive care measures—including growth factor support and better infection prevention and management—likely resulted in delivering treatment more safely and completely [16, 20, 21]. Secondly, treatment advances in the relapsed/refractory setting might also have contributed to recent improvements in survival. However, treatment data in the relapsed/refractory setting were as yet not available in our study. Lastly, stage migration might have caused artificial survival gains in more recent periods. Nevertheless, we are confident that stage migration only marginally affected the survival estimates because survival improvements were objectified for the entire cohort where all stages were included wholly.

The comparatively modest survival gains beyond the introduction of rituximab, along with an apparent lack of a plateau in the relative survival curve in most patient subsets, should stimulate initiatives across various treatment lines, age segments, and biological subgroups to increase cure rates and reduce late treatment-related excess mortality.

### Strengths and limitations

Our study has several strengths, including using a well-established and long-running cancer registry with comprehensive data available on primary therapy. Using a nationwide registry averts selection bias that is generally deep-rooted in clinical trials, thereby enabling a generalization of our results. Also, the NCR ascertains more detailed data on primary therapy for patients diagnosed as from 2014, allowing to assess temporal trends in applying different R-CHOP regimens in modern times. This type of information is usually lacking in nationwide cancer registries.

Limitations of our study include the lack of information on the cause of death throughout the entire registry and the lack of detailed information on primary therapy and clinical and lymphoma-related factors throughout most of the registry (1989–2013). Also, second and subsequent treatment lines were as yet not available in the NCR; hence, impeding the calculation of progression-free survival and assessing treatment strategies in the relapsed/refractory setting. Nevertheless, the NCR is restructured to allow for the ascertainment of treatment beyond 1-year post-diagnosis for patients diagnosed as of 2014 via retrospective medical records review. Thus, in the future, with prolonged follow-up, it will be possible to characterize the contemporary treatment landscape in the relapsed/refractory setting, rendering a population-based variant of the SCHOLAR-1 study [61]. Also, it allows addressing the comparative therapeutic value of treatment strategies in clinical practice across various therapy lines; for example, the effectiveness of 6x R-CHOP versus 6x R-CHOP + 2 R for the first-line treatment of DLBCL. Lastly, the effect of comparatively minor revisions in the classification system of lymphomas is unknown; however, given the low frequency of the alternative diagnoses, it is expected to be marginal.

## Concluding remarks

In this nationwide, population-based study, relative survival among adult DLBCL patients across various subgroups of age and stage continues to increase, albeit modestly, in an almost two-decade-old era where rituximab-containing chemotherapy is still the standard first-line therapy. Notwithstanding this encouraging finding, excess mortality remains an issue in modern times, particularly in older-aged patients. These findings underscore the importance of optimizing current treatment strategies and the need for novel therapies in DLBCL.

## Supplementary information

ONLINE APPENDIX
