# Supplementary material for: Time trends in primary therapy and relative survival of diffuse large B-cell lymphoma by stage: a nationwide, population-based study in the Netherlands, 1989–2018
Source: Blood Cancer J. 2022 Mar 9;12(3):38. doi: 10.1038/s41408-022-00637-1 (PMC8907354; doi:10.1038/s41408-022-00637-1)

**ONLINE APPENDIX**

**Title**

Time trends in primary therapy and relative survival of diffuse large B-cell lymphoma by stage: a nationwide, population-based study in the Netherlands, 1989-2018

**Authors and affiliations**

Müjde Durmaz,^1^ Otto Visser,^2^  Eduardus F.M. Posthuma,^3,4^ Rolf E. Brouwer,^3^ Djamila E. Issa,^5^ Daphne de Jong,^6^ King H. Lam^7^, Nicole M.A. Blijlevens,^8^ Josée M. Zijlstra,^9^ Martine E.D. Chamuleau,^9^ Pieternella J. Lugtenburg,^10^ Marie José Kersten,^11^ Avinash G. Dinmohamed^1,9,11,12^

^1^Department of Research and Development, Netherlands Comprehensive Cancer Organisation (IKNL), Utrecht, the Netherlands; ^2^Department of Registration, Netherlands Comprehensive Cancer Organisation (IKNL), Utrecht, The Netherlands; ^3^Department of Internal Medicine, Reinier de Graaf Gasthuis, Delft, The Netherlands; ^4^Department of Hematology, Leiden University Medical Center, Leiden, The Netherlands; ^5^Department of Internal Medicine, Jeroen Bosch Hospital, Den Bosch, The Netherlands; ^6^Amsterdam UMC, Department of Pathology, Vrije Universiteit Amsterdam, Cancer Center Amsterdam, Amsterdam, The Netherlands; ^7^Department of Pathology, Erasmus MC Cancer Institute, Rotterdam, The Netherlands; ^8^Department of Hematology, Radboud University Medical Center, Nijmegen, The Netherlands; ^9^Amsterdam UMC, Vrije Universiteit Amsterdam, Cancer Center Amsterdam, Department of Hematology, Amsterdam, The Netherlands; ^10^Department of Hematology, Erasmus MC, Cancer Institute, University Medical Center Rotterdam, Rotterdam, The Netherlands; ^11^Amsterdam UMC, University of Amsterdam, Department of Hematology, Amsterdam, The Netherlands; ^12^Department of Public Health, Erasmus MC, University Medical Center Rotterdam, Rotterdam, The Netherlands

**Supplemental Table 1**

**Supplemental Table 1.** Incidence rates of patients diagnosed with diffuse large B-cell lymphoma in the Netherlands, 1989-2018.

| **Stage** | **Characteristics** | **Calendar period** | | | **Total** |
| --- | --- | --- | --- | --- | --- |
|  |  | **1989-2002** | **2003-2010** | **2011-2018** |  |
| **Total** | **Overall** | 4.73 | 5.30 | 5.36 | 5.05 |
|  | **Sex** |  |  |  |  |
|  | Male | 5.59 | 6.28 | 6.44 | 6.00 |
|  | Female | 3.87 | 4.33 | 4.29 | 4.10 |
|  | **Age, years** |  |  |  |  |
|  | 20-64 | 3.46 | 4.09 | 4.36 | 3.87 |
|  | 65-74 | 18.22 | 20.77 | 22.16 | 19.95 |
|  | ≥75 | 28.95 | 36.31 | 34.87 | 32.49 |
| **I** | **Overall** | 1.33 | 1.29 | 1.02 | 1.24 |
|  | **Sex** |  |  |  |  |
|  | Male | 1.57 | 1.55 | 1.25 | 1.48 |
|  | Female | 1.10 | 1.03 | 0.79 | 1.00 |
|  | **Age. years** |  |  |  |  |
|  | 20-64 | 1.01 | 0.99 | 0.82 | 0.95 |
|  | 65-74 | 4.83 | 4.81 | 3.85 | 4.56 |
|  | ≥75 | 8.04 | 9.22 | 6.91 | 8.06 |
| **II-IV** | **Overall** | 2.99 | 3.83 | 4.21 | 3.54 |
|  | **Sex** |  |  |  |  |
|  | Male | 3.53 | 4.51 | 5.03 | 4.19 |
|  | Female | 2.45 | 3.14 | 3.39 | 2.89 |
|  | **Age. years** |  |  |  |  |
|  | 20-64 | 2.26 | 3.03 | 3.48 | 2.79 |
|  | 65-74 | 11.66 | 15.41 | 17.89 | 14.32 |
|  | ≥75 | 16.97 | 24.58 | 25.97 | 21.40 |
| All overall and sex-specific incidence rates are age-adjusted to the European standard population and expressed per 100,000 person-years. | | | | | |

**Supplemental Table 2**

**Supplemental Table 2.** Patient characteristics for the period 2014-2018.

| **Characteristics** | **Disease stage** | | | | | | **Total** | |
| --- | --- | --- | --- | --- | --- | --- | --- | --- |
|  | **I** | | **II-IV** | | **Unknown** | |  |  |
|  | **N** | **(%)** | **N** | **(%)** | **N** | **(%)** | **N** | **(%)** |
| **Total no. of patients (row %)** | **1,103** | **(18)** | **5,034** | **(80)** | **160** | **(3)** | **6,297** | **(100)** |
| **Year of diagnosis (row %)** |  |  |  |  |  |  |  |  |
| 2014 | 245 | (20) | 945 | (77) | 41 | (3) | 1,231 | (20) |
| 2015 | 223 | (18) | 961 | (79) | 29 | (2) | 1,213 | (19) |
| 2016 | 228 | (17) | 1,054 | (80) | 32 | (2) | 1,314 | (21) |
| 2017 | 177 | (14) | 1,034 | (84) | 27 | (2) | 1,238 | (20) |
| 2018 | 230 | (18) | 1,040 | (80) | 31 | (2) | 1,301 | (21) |
| **Sex** |  |  |  |  |  |  |  |  |
| Male | 650 | (18) | 2,876 | (80) | 78 | (2) | 3,604 | (57) |
| Female | 453 | (17) | 2,158 | (80) | 82 | (3) | 2,693 | (43) |
| **Age, years** |  |  |  |  |  |  |  |  |
| Median (IQR) | 70 (59-78) | | 70 (60-77) | | 82 (74-88) | | 70 (60-78) | |
| 18-64 | 397 | (36) | 1,768 | (35) | 17 | (11) | 2,182 | (35) |
| 65-74 | 311 | (28) | 1,568 | (31) | 25 | (16) | 1,904 | (30) |
| ≥75 | 395 | (36) | 1,698 | (34) | 118 | (74) | 2,211 | (35) |
| **WHO performance status** |  |  |  |  |  |  |  |  |
| 0 | 421 | (38) | 1,302 | (26) | 10 | (6) | 1,733 | (28) |
| 1 | 170 | (15) | 793 | (16) | 11 | (7) | 974 | (15) |
| 2 | 41 | (4) | 317 | (6) | 19 | (12) | 377 | (6) |
| 3 or 4 | 18 | (2) | 199 | (4) | 17 | (11) | 234 | (4) |
| Unknown | 453 | (41) | 2,423 | (48) | 112 | (70) | 2,988 | (47) |
| **Ann Arbor stage** |  |  |  |  |  |  |  |  |
| 1 | 1,103 | (100) | - | - | - | - | 1,103 | (18) |
| 2 | - | - | 1,124 | (22) | - | - | 1,124 | (18) |
| 3 | - | - | 1,139 | (23) | - | - | 1,139 | (18) |
| 4 | - | - | 2,771 | (55) | - | - | 2,771 | (44) |
| Unknown | - | - | - | - | 160 | (100) | 160 | (3) |
| **Elevated LDH** |  |  |  |  |  |  |  |  |
| No | 819 | (74) | 1,859 | (37) | 46 | (29) | 2,724 | (43) |
| Yes | 235 | (21) | 3,049 | (61) | 80 | (50) | 3,364 | (53) |
| **>1 extranodal localizations** |  |  |  |  |  |  |  |  |
| No | 1,088 | (99) | 3,350 | (67) | 79 | (49) | 4,517 | (72) |
| Yes | 13 | (1) | 1,682 | (33) | 70 | (44) | 1,765 | (28) |
| **IPI score** |  |  |  |  |  |  |  |  |
| 0 or 1 (low) | 509 | (46) | 508 | (10) | 0 | (0) | 1,017 | (16) |
| 2 (low intermediate) | 98 | (9) | 658 | (13) | 0 | (0) | 756 | (12) |
| 3 (high intermediate) | 25 | (2) | 756 | (15) | 0 | (0) | 781 | (12) |
| 4 or 5 (high) | 0 | (0) | 1,086 | (22) | 8 | (5) | 1,094 | (17) |
| Undetermined | 471 | (43) | 2,026 | (40) | 152 | (95) | 2,649 | (42) |
| Abbreviations: WHO, World Health Organisation; IPI, International Prognostic Index; LDH, Lactate Dehydrogenase and IQR, InterQuartile Range. | | | | | | | | |

**Supplemental Figure 1**

**Supplemental Figure 1.** Incidence rates of patients with stage I and stage II-IV DLBCL in the Netherlands according to sex and age at diagnosis for the three calendar periods, 1989-2018.


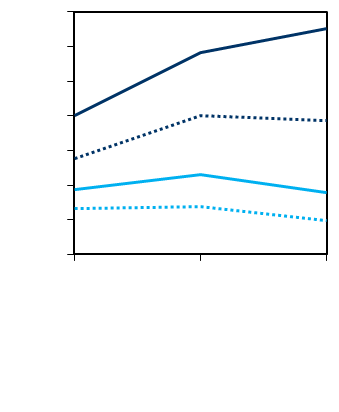

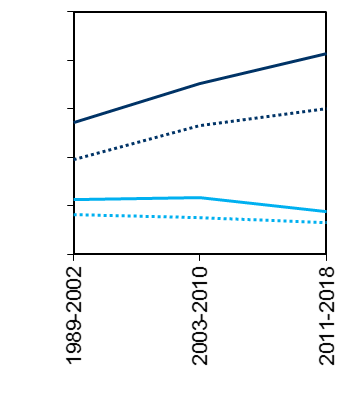

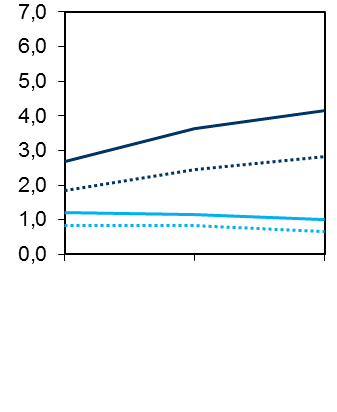


**Year of diagnosis**

**Incidence per 100,000**

stage I, female

stage I, male

stage II-IV, female

stage II-IV, male

**B: 65-74 years**

**A: 20-64 years**

**C: ≥75 years**

**Supplemental Figure 2**

**Supplemental Figure 2.** Age-specific incidence rates of patients with stage I and stage II-IV DLBCL in the Netherlands according to sex, 1989-2018.

**Supplemental Figure 3**

**Supplemental Figure 3.** Primary treatment of patients with stage I in the Netherlands according to age at diagnosis and calendar year of diagnosis, 2014-2018. The proportion of patients receiving a particular treatment within a specific calendar year and age group are presented in the column below. Abbreviations: R, rituximab; CHOP, cyclophosphamide, doxorubicin, vincristine, and prednisone and RT, radiotherapy.

**Stage I disease**


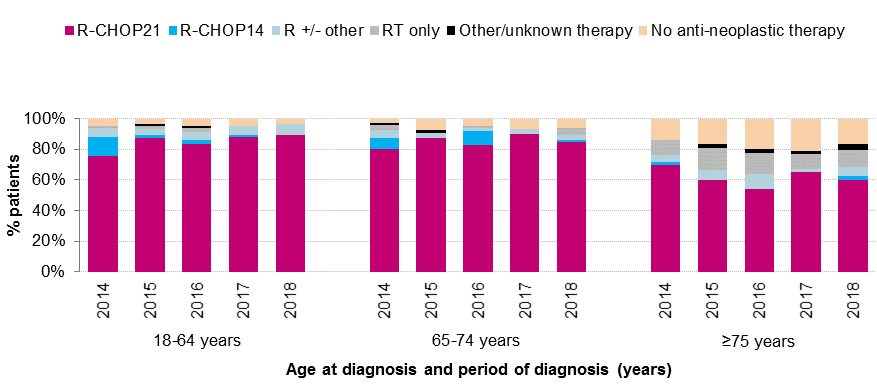


N=92

N=83

N=85

N=52

N=70

N=54

N=63

N=59

N=65

N=83

N=86

N=80

N=66

N=82

N=83

**
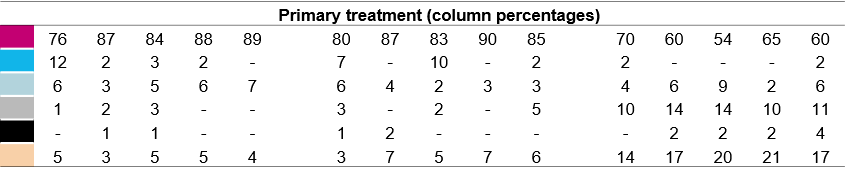
**

**Supplemental Figure 4**

**Supplemental Figure 4.** Primary treatment of patients with stage II-IV in the Netherlands according to age at diagnosis and calendar year of diagnosis, 2014-2018. The proportion of patients receiving a particular treatment within a specific calendar year and age group are presented in the column below. Abbreviations: R, rituximab; CHOP, cyclophosphamide, doxorubicin, vincristine, and prednisone and RT, radiotherapy.

**Stage II/IV disease**


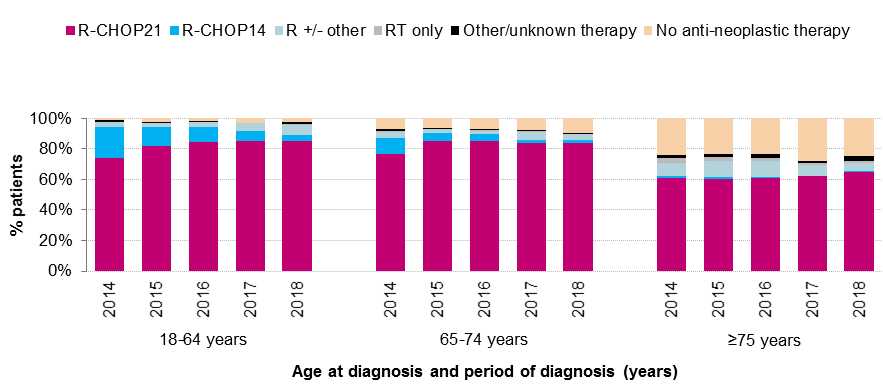


N=326

N=318

N=361

N=327

N=278

N=307

N=317

N=347

N=319

N=341

N=336

N=376

N=360

N=355

N=366


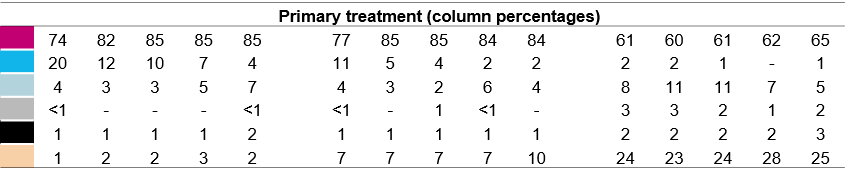

Supplement: Supplementary file 1 — ONLINE APPENDIX [file 41408_2022_637_MOESM1_ESM.docx]
